# Right ventricle to pulmonary artery coupling in patients with primary mitral regurgitation: association with outcome

**DOI:** 10.1093/ehjci/jeag037

**Published:** 2026-01-31

**Authors:** Nadeem Elmasry, Pilar Lopez Santi, Bart J J Velders, Hoi Wai Wu, Meindert Palmen, Jeroen J Bax, Mohamed Hussein, Mireille Bherer, Philippe Pibarot, Nina Ajmone Marsan

**Affiliations:** Department of Cardiology, Leiden University Medical Center, Albinusdreef 2, Leiden 2333, The Netherlands; Department of Cardiology, Ain Shams University, Cairo, Egypt; Department of Cardiology, Leiden University Medical Center, Albinusdreef 2, Leiden 2333, The Netherlands; Department of Cardiothoracic Surgery, Leiden University Medical Center, Leiden, The Netherlands; Department of Cardiology, Leiden University Medical Center, Albinusdreef 2, Leiden 2333, The Netherlands; Department of Cardiothoracic Surgery, Leiden University Medical Center, Leiden, The Netherlands; Department of Cardiology, Leiden University Medical Center, Albinusdreef 2, Leiden 2333, The Netherlands; Department of Cardiology, Turku Heart Center, University of Turku and Turku University Hospital, Turku, Finland; Department of Cardiology, Ain Shams University, Cairo, Egypt; Department of Cardiology, Institut Universitaire de Cardiologie et de Pneumologie de Québec – Université Laval/Quebec Heart and Lung Institute – Laval University, Quebec city, Quebec, Canada; Department of Cardiology, Institut Universitaire de Cardiologie et de Pneumologie de Québec – Université Laval/Quebec Heart and Lung Institute – Laval University, Quebec city, Quebec, Canada; Department of Cardiology, Leiden University Medical Center, Albinusdreef 2, Leiden 2333, The Netherlands

In patients with significant primary mitral regurgitation (PMR), both elevated pulmonary pressure and right ventricular (RV) dysfunction are associated with adverse outcomes.^1,2^ However, the adaptation of the RV to increased pulmonary pressures can vary, even within ranges of RV function considered normal. The concept of coupling the RV to pulmonary artery (PA) has therefore been introduced to describe the match between the RV contractility and the opposing afterload,^3^ and the aim of this study was to evaluate the association of RV–PA coupling with outcome in PMR patients undergoing mitral valve (MV) surgery.

RV–PA coupling was measured shortly before surgery as the ratio of tricuspid annular plane systolic excursion (TAPSE) to systolic pulmonary artery pressure (sPAP) in 485 patients with PMR and elevated sPAP (≥30 mmHg) at two tertiary centres: Leiden University Medical Center (the Netherlands), and Québec Heart and Lung Institute (Canada). Data were retrospectively analysed in the echo-core lab of both centres. Patients who underwent previous cardiac surgery, and those with unavailable TAPSE or sPAP were excluded. The study outcome was all-cause mortality.

During a median follow-up of 8.3 (Interquartile range [IQR] 4.3–12.9) years, 94 (19.4%) patients died. Spline curve analysis identified an age and sex adjusted TAPSE/sPAP cutoff of 0.48 mm/mmHg corresponding to a hazard ratio (HR) of 1 (*Figure 1A*). According to this cutoff, 304 (62.7%) patients were classified as RV–PA coupling group (ratio ≥0.48), while 181 (37.3%) were classified as RV–PA uncoupling group (ratio <0.48). Patients in the RV–PA uncoupling group were significantly older (mean age 70 vs. 65 years), more symptomatic (88% vs. 69%) and had higher rates of atrial fibrillation (AF) (54% vs. 31%) and diuretic use (63% vs. 38%), *P* < 0.001 for all. Furthermore, they had lower left ventricular ejection fraction (mean 62.2% vs. 64.1%; *P* = 0.027), larger left atrial volume index (median 57.1 vs. 52.0 mL/m^2^; *P* = 0.032), and higher prevalence of significant tricuspid regurgitation (TR > Grade II 20% vs. 6%, *P* < 0.001) and therefore concomitant tricuspid valve repair (65% vs. 44%, *P* < 0.001).

**Figure 1:**
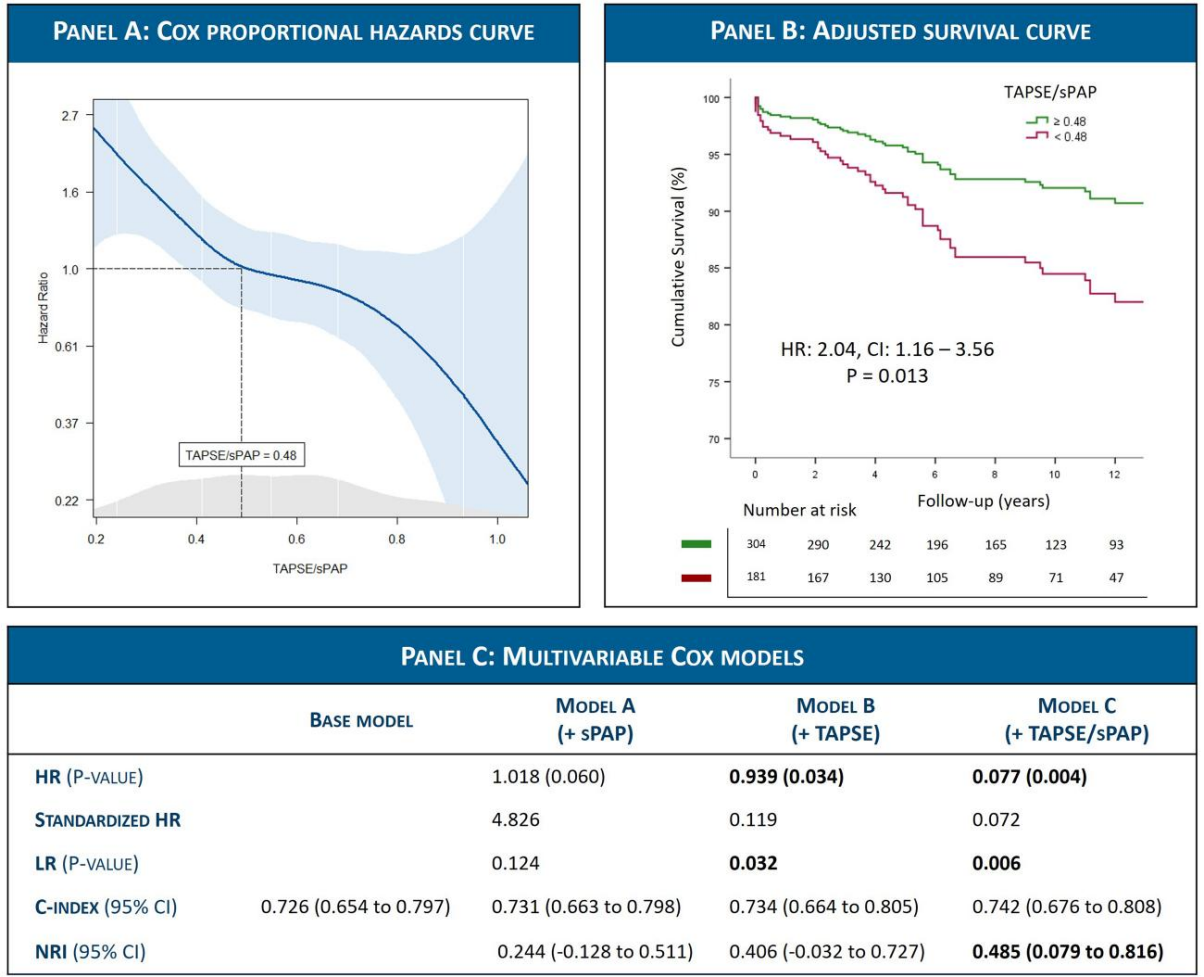
(*A*) Determination of optimal age and sex adjusted TAPSE/sPAP cutoff using Cox proportional hazards. The solid line represents the hazard ratio for all-cause mortality, and the shaded areas represent the 95% CI. Values <0.48 were associated with hazard ratio ≥ 1. (*B*) Adjusted survival curve demonstrating the difference in survival between the RV–PA coupling vs. uncoupling groups at a cutoff of 0.48 mm/mmHg, after adjustment for significant covariates: Age, symptoms (NYHA Classes II–IV), atrial fibrillation, glomerular filtration rate, mitral annular e′, and significant TR > Grade II. (*C*) Cox regression models for all-cause mortality. The baseline model included age, NYHA Class ≥ II, eGFR, atrial fibrillation, average e′, and significant TR > Grade II. The variables sPAP, TAPSE, and TAPSE/sPAP were added as continuous variables separately to the baseline model to form Models A, B, and C, respectively. CI, confidence interval; HR, hazard ratio; LR, likelihood ratio; NRI, net reclassification improvement; NYHA, New York Heart Association; SPAP, systolic pulmonary artery pressure; TAPSE, tricuspid annular plane systolic excursion; TR, tricuspid regurgitation.

On univariable analysis, TAPSE (HR = 0.92; *P* < 0.001), sPAP (HR = 1.02; *P* = 0.006), and TAPSE/sPAP (HR = 0.07; *P* < 0.001) as continuous variables were all significantly associated with all-cause mortality. To avoid collinearity on multivariable analysis, each of these parameters was added separately to a baseline model including age, symptoms [New York Heart Association (NYHA) Class II–IV], atrial fibrillation, glomerular filtration rate (eGFR), mitral annular e’, and significant TR > Grade II (the only variables significantly associated with the endpoint at univariable analysis). Both TAPSE (HR = 0.94; *P* = 0.034) and TAPSE/sPAP (HR = 0.08; *P* = 0.004) remained independently associated with mortality in their respective models, whereas sPAP did not (HR = 1.02; *P* = 0.060). Furthermore, TAPSE/sPAP was also an independent predictor when added as a dichotomous variable to the baseline model, at a cutoff of 0.48 (HR = 2.04; *P* = 0.013) (*Figure 1B*). This cutoff was further internally validated by bootstrap analysis of the Cox model (1000 samples), where it remained independently associated with the outcome [regression coefficient =0.71; 95% confidence interval (CI) = 0.12–1.38; *P* = 0.021].

The predictive value of adding TAPSE/sPAP vs. TAPSE or sPAP alone to the baseline model was further compared (*Figure 1C*). Adding the TAPSE/sPAP ratio resulted in a significant net reclassification improvement 0.485 (95% CI: 0.079–0.816), while TAPSE or sPAP alone did not.

A subgroup analysis was performed for patients with only mildly elevated sPAP (30–50 mmHg, *n* = 347, 71.5%), in whom TAPSE showed similar values as in patients with markedly elevated sPAP > 50 mmHg (22.9 vs. 22.6 mm, *P* = 0.438). Kaplan–Meier analysis performed in this subgroup showed that RV–PA uncoupling was also significantly associated with all-cause mortality (*P* < 0.001). In addition, in a model adjusted for age, symptoms, AF, and eGFR, RV–PA uncoupling was independently associated with mortality (HR = 1.82; 95% CI: 1.02–3.25; *P* = 0.041).

To our knowledge, this is the first study to investigate the prognostic impact of RV–PA coupling in patients with PMR and elevated pulmonary pressures undergoing MV surgery. The ratio of TAPSE/sPAP was used as a non-invasive coupling index since TAPSE is easily obtainable and highly reproducible, with previous studies showing it significantly correlates with invasive coupling parameters and outcome across a range of cardiovascular pathologies.^3,4^ Our study focused on patients who presented with elevated pulmonary pressures, where RV dysfunction has been shown to further risk stratify these patients and provide an additional prognostic value.^2,5^ Limitations of this study include the need for further validation of the proposed cutoff in different PMR cohorts, the unavailability of sPAP estimation in patients with incomplete TR envelope, and the lack of detailed causes of mortality. In addition, other parameters of RV–PA coupling using fractional area change or RV strain were not systematically available, as well as left atrial strain for comparative analyses.

In conclusion, RV–PA coupling represented by the ratio of TAPSE/sPAP was independently associated with all-cause mortality in PMR patients undergoing valve surgery, at a proposed cutoff of <0.48 mm/mmHg, and provided better risk stratification than TAPSE or sPAP alone.

## Data Availability

Anonymized data underlying this study are available upon reasonable request to the corresponding author. Access may be subject to ethical and institutional approval to ensure compliance with data protection regulations.

## References

[1] Praz F, Borger MA, Lanz J, Marin-Cuartas M, Abreu A, Adamo M et al 2025 ESC/EACTS guidelines for the management of valvular heart disease. Eur Heart J 2025;46(44):4635–4736. 40878295 10.1093/eurheartj/ehaf194

[2] van Wijngaarden AL, Mantegazza V, Hiemstra YL, Volpato V, van der Bijl P, Pepi M et al Prognostic impact of extra-mitral valve cardiac involvement in patients with primary mitral regurgitation. JACC Cardiovasc Imaging 2022;15:961–70. 35033499 10.1016/j.jcmg.2021.11.009

[3] Guazzi M, Bandera F, Pelissero G, Castelvecchio S, Menicanti L, Ghio S et al Tricuspid annular plane systolic excursion and pulmonary arterial systolic pressure relationship in heart failure: an index of right ventricular contractile function and prognosis. Am J Physiol Heart Circ Physiol 2013;305:H1373–81. 23997100 10.1152/ajpheart.00157.2013

[4] Cahill TJ, Pibarot P, Yu X, Babaliaros V, Blanke P, Clavel MA et al Impact of right ventricle-pulmonary artery coupling on clinical outcomes in the PARTNER 3 trial. JACC Cardiovasc Interv 2022;15:1823–33. 36137685 10.1016/j.jcin.2022.07.005

[5] Bohbot Y, Essayagh B, Benfari G, Bax JJ, Le Tourneau T, Topilsky Y et al Prognostic implications of right ventricular dysfunction in severe degenerative mitral regurgitation. J Am Heart Assoc 2025;14:e036206. 39692024 10.1161/JAHA.124.036206 PMC12054403

